# Cerebellar and basal ganglia structural connections in humans: Effect of aging and relation with memory and learning

**DOI:** 10.3389/fnagi.2023.1019239

**Published:** 2023-01-26

**Authors:** Vineeth Radhakrishnan, Cecile Gallea, Romain Valabregue, Syam Krishnan, Chandrasekharan Kesavadas, Bejoy Thomas, Praveen James, Ramshekhar Menon, Asha Kishore

**Affiliations:** ^1^Comprehensive Care Centre for Movement Disorders, Department of Neurology, Sree Chitra Tirunal Institute of Medical Sciences and Technology, Thiruvananthapuram, India; ^2^INSERM, CNRS, Paris Brain Institute, Sorbonne Université, Paris, France; ^3^Department of Imaging Sciences and Interventional Radiology, Sree Chitra Tirunal Institute of Medical Sciences and Technology, Thiruvananthapuram, India; ^4^Department of Neurology, Sree Chitra Tirunal Institute for Medical Sciences and Technology, Thiruvananthapuram, India; ^5^Parkinson and Movement Disorder Centre, Department of Neurology, Aster Medcity, Kochi, India

**Keywords:** aging, memory, learning, cerebellum, basal ganglia, tractography, connectivity, diffusion

## Abstract

**Introduction:**

The cerebellum and basal ganglia were initially considered anatomically distinct regions, each connected *via* thalamic relays which project to the same cerebral cortical targets, such as the motor cortex. In the last two decades, transneuronal viral transport studies in non-human primates showed bidirectional connections between the cerebellum and basal ganglia at the subcortical level, without involving the cerebral cortical motor areas. These findings have significant implications for our understanding of neurodevelopmental and neurodegenerative diseases. While these subcortical connections were established in smaller studies on humans, their evolution with natural aging is less understood.

**Methods:**

In this study, we validated and expanded the previous findings of the structural connectivity within the cerebellum-basal ganglia subcortical network, in a larger dataset of 64 subjects, across diﬀerent age ranges. Tractography and fixel-based analysis were performed on the 3 T diﬀusion-weighted dataset using Mrtrix3 software, considering fiber density and cross-section as indicators of axonal integrity. Tractography of the well-established cerebello-thalamo-cortical tract was conducted as a control. We tested the relationship between the structural white matter integrity of these connections with aging and with the performance in diﬀerent domains of Addenbrooke’s Cognitive Examination.

**Results:**

Tractography analysis isolated connections from the dentate nucleus to the contralateral putamen *via* the thalamus, and reciprocal tracts from the subthalamic nucleus to the contralateral cerebellar cortex *via* the pontine nuclei. Control tracts of cerebello-thalamo-cortical tracts were also isolated, including associative cerebello-prefrontal tracts. A negative linear relationship was found between the fiber density of both the ascending and descending cerebellum-basal ganglia tracts and age. Considering the cognitive assessments, the fiber density values of cerebello-thalamo-putaminal tracts correlated with the registration/learning domain scores. In addition, the fiber density values of cerebello-frontal and subthalamo-cerebellar (Crus II) tracts correlated with the cognitive assessment scores from the memory domain.

**Conclusion:**

We validated the structural connectivity within the cerebellum-basal ganglia reciprocal network, in a larger dataset of human subjects, across wider age range. The structural features of the subcortical cerebello-basal ganglia tracts in human subjects display age-related neurodegeneration. Individual morphological variability of cerebellar tracts to the striatum and prefrontal cortex was associated with diﬀerent cognitive functions, suggesting a functional contribution of cerebellar tracts to cognitive decline with aging. This study oﬀers new perspectives to consider the functional role of these pathways in motor learning and the pathophysiology of movement disorders involving the cerebellum and striatum.

## 1. Introduction

For a long time, basal ganglia (BG) and cerebellum (CB) were considered anatomically and functionally distinct subcortical structures, each involved in specific types of learning (Doya, 2000), namely reinforcement and supervised learning (Kawato and Gomi, 1992; Schultz et al., 1997; Doya, 2000; O’Doherty et al., 2003). The two structures project to cortical areas *via* separate thalamic nuclei forming the striato-thalamo-cortical (STC) and cerebello-thalamocortical (CTC) loops (Kemp and Powell, 1971; Allen et al., 1978; Asanuma et al., 1983; Sakai et al., 1996). Abnormal engagement of these loops in diseases involving BG and/or CB results in different behavioral impairments. For instance, Parkinson’s disease (PD) with dysregulation of the striatal dopaminergic pathway shows impaired reinforcement learning (Voon et al., 2010), while ataxic patients with structural abnormalities of the CB show decreased error-based (supervised) learning during sensorimotor adaptation (Panouillères et al., 2017). These traditional perspectives were challenged by neuroanatomical studies in primates that demonstrated reciprocal connections between BG and CB through thalamic and pontine structures without involving cortical cerebral areas, raising doubts about such clear functional dissociation. Dense disynaptic projections were demonstrated between the dentate nucleus (DN) of the CB and the putamen *via* the central-lateral nucleus of the thalamus and between the subthalamic nucleus (STN) and the cerebellar cortex *via* the pontine nucleus (Hoshi et al., 2005; Bostan et al., 2010; Bostan and Strick, 2018). A pathological interaction between the two structures is also suspected in movement disorders such as PD (Wu and Hallett, 2013; Kishore et al., 2014; Kishore and Popa, 2014), dystonia (Sadnicka et al., 2012; Kaji et al., 2018), Tourette’s syndrome and psychiatric disorders such as attention-defcit/hyperactivity disorder, and schizophrenia (Strick et al., 2009; Maia and Frank, 2011; O’Halloran et al., 2012). However, whether the morphology and the functional contribution of CB-BG reciprocal connections are affected by normal aging is not well-delineated.

Investigating anatomical connections within the CB-BG network in the human brain is possible *in-vivo* with non-invasive diffusion-weighted imaging (DWI) and tractography. These techniques can measure connectivity strength, i.e., the probability of connection based on the density of streamlines from a seed to a target region. In 12 participants, an exploratory study quantified the connectivity strength of the cerebellar output pathways involving the DN, respectively, to the caudate (12%), the putamen (9%), and the pallidum (11%; Pelzer et al., 2013). In 15 participants, another study used constrained spherical deconvolution (CSD) capable of resolving the crossing, kissing, or branching white matter bundles (Milardi et al., 2016). This study established contralateral tracts from the DN to the thalamus as in the primate studies and also proposed the presence of contralateral and ipsilateral tracts from DN to the thalamus, and from STN to the CB cortex (Hoshi et al., 2005). Given the inter-individual variability of human anatomy, studies involving a larger number of participants are needed to validate these exploratory findings.

While the cognitive functions of the BG are well-recognized (Haber, 2003), CB is more recently considered as a hub that regulates various non-motor functions (Hoche et al., 2018; Schmahmann, 2019). Descending and ascending connections between the CB and the cerebral cortical areas involving the prefrontal cortex have been described and corroborate the role of the CB in modulating cognitive behavior (Parker et al., 2014; Jobson et al., 2021). Lobules I–VI and VIII are involved in sensorimotor tasks, whereas the Crus II lobule is primarily associated with non-motor functions (Stoodley et al., 2012, 2020), especially social mentalizing, and emotional self-experiences (Van Overwalle et al., 2020a, b) language, emotions, and working memory (Habas et al., 2009; Stoodley and Schmahmann, 2009). The functional contribution of the CB-BG connections is often inferred and not directly assessed. Recent studies showed that both CB and BG contribute to associative and reward-based learning, suggesting a physiological interaction between them (Wu and Hallett, 2013; Kishore et al., 2014; Kishore and Popa, 2014).

Age-related degeneration of white matter tracts such as CB-BG interconnections could result in the decline of functions, including movement and cognition. Age-related degeneration of white matter tracts affects cortical brain areas and subcortical structures including BG (Raz et al., 2003; Koikkalainen et al., 2007; Zwirner et al., 2016). Age-related changes in water diffusivity in the white matter of the middle cerebellar peduncles were observed in several studies (Cox et al., 2016; Coelho et al., 2021). Recent technical advances of DTI provide an opportunity to investigate the evolution of the integrity of white matter connections with age. For instance, the “disconnection hypothesis” suggests that age-related cognitive decline is linked to brain structural changes, i.e., the alteration of white matter tracts between cortical regions can lead to a decline in cognitive performance (Bennett and Madden, 2014; Fjell et al., 2016). However, the specific contribution of cerebellar connections to this process is unclear. As healthy aging itself influences the morphological characteristics of white matter tracts, including cerebellar tracts, its impact on CB-BG connections should be known to better understand the changes in pathological conditions. Since aging affects cognitive functions (Bendlin et al., 2010; Coelho et al., 2021), we hypothesized that aging will affect cerebellar pathways (including the CB-BG connections) proportionally to cognitive abilities.

In the present study, we aimed to (i) validate and expand the imaging evidence of the presence of the anatomical tracks linking CB and BG as reported in non-human primates (Hoshi et al., 2005; Bostan et al., 2010) and (ii) evaluate age-related changes in the morphology of CB-BG reciprocal connections and its relation to specific cognitive domains. We enrolled 64 human healthy subjects across a wide age range (30–80 yrs.). We applied DWI with CSD.

We evaluated the age-related neurodegeneration on the CB-BG reciprocal white matter tracts using the fixel-based analysis (FBA) technique. This novel model for diffusion MRI analysis is optimized to isolate crossing or kissing fibers and allows evaluating axonal parameters along a tract, namely fiber density (FD, reflecting intra-axonal volume), fiber cross-section (FC, reflecting the area occupied by the axons), and a combination of FD and FC, namely FDC (Raffelt et al., 2015). In addition, we tested the association between the individual characteristics of FBA metrics and the measures of specific cognitive performance. Since the cerebellum and prefrontal cortices are involved in learning and retention (Elhalal et al., 2014), cognitive assessments in these domains were used to explore their relationship with the FBA metrics.

## 2. Materials and methods

### 2.1. Subjects and neuropsychological testing

Sixty-four healthy volunteers (HV; mean age:55.69 ± 9.96 years, M/F:34/30, age range:30–80 years) with no history of neurological or psychiatric illness and formal education > 6 years were recruited for the study over 3 years from a single center (Sree Chitra Tirunal Institute for Medical Sciences and Technology, India). The MRI scans were screened by a radiologist for any structural non-symptomatic lesions. The subjects were recruited *via* notification exhibited at the hospital campus. Among these 64 participants, 20 subjects (mean age:56.8 ± 8.7 years, age range: 41–66 years) with a Clinical Dementia Rating (CDR) score of 0, underwent neuropsychological battery tests assessing global cognitive score *via* vernacular (Malayalam, a south Indian language) adaptation of Addenbrooke’s Cognitive Examination battery (ACE-M) along with Rey Auditory Verbal Learning Test (RAVLT; Mathuranath et al., 2004; Menon et al., 2014). Learning and retention were assessed *via* distinct domains of ACE neuropsychological analysis: ACE-M-Reg-24 (Registration/learning: 24 point scale; registration of 3 words = 3 points; 3-trial learning of an address = 21 points), ACE-M-Recall [10 point scale (recall of 3 words, each after a delay of 5 min = 3 points; recall of address = 7 points)], along with the total score of ACE-M. Other retention scores included the delayed recall and total scores of RAVLT.

### 2.2. Ethics statement

All subjects provided written informed consent according to the declaration of Helsinki and the study was approved by the Institutional Ethics Committee (SCT/IEC/816/OCTOBER-2015).

### 2.3. MRI data acquisition

Structural and diffusion MRI data were acquired in a 3-tesla scanner (GE MEDICAL SYSTEMS, Discovery MR750w, Chicago, Illinois, United States) using a 32-channel, phased-array head coil designed for parallel imaging. A high-resolution 3D, T1-weighted, fast spoiled, gradient-echo sequence (TR = 7.924 ms, TE = 2.984 ms, Flip angle = 12, matrix = 256 × 256, 172 sections of 1 mm each) and diffusion-weighted MRI (dMRI) of single-shot echo-planar spin-echo sequence with 64 directions (TR = 9,000 ms, TE = 99.8 ms, matrix = 256 × 256, b = 0 and 1,000 s/mm^2^, 57 slices of 2 mm thick) were acquired for each subject.

### 2.4. Tractography

**Figure 1:**
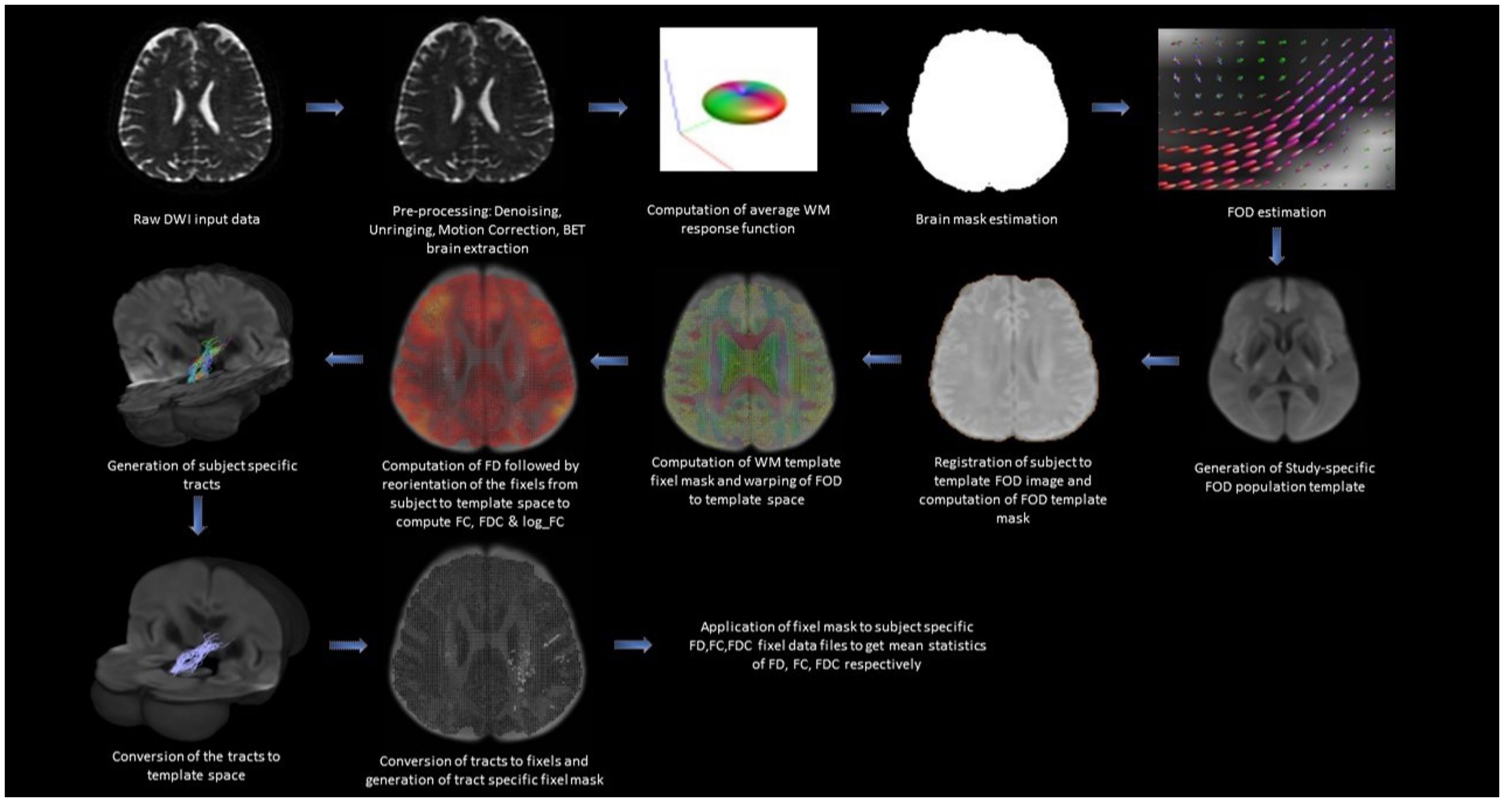
Figure showing the steps involved in the generation of tract-specific FBA metrics.

The tractography analysis was performed using the Mrtrix3 toolbox (Tournier et al., 2019) and the functions in the FSL toolkit as shown in Figure 1. Standard pre-processing was performed including denoising using the Marchenko-Pastur Principal Component Analysis (MP-PCA; Veraart et al., 2016; Cordero-Grande et al., 2019) followed by Gibbs ringing removal (Kellner et al., 2015), and Eddy current correction (Smith et al., 2004). The non-brain tissues were removed using Brain Extraction Tool (BET; Smith, 2002; Smith et al., 2004). We estimated the response function from the brain using the *dwi2response* function with the “dhollander” algorithm (Tournier et al., 2004). We then estimated the fiber orientation distribution (FOD) based on eighth-order CSD using the *dwi2fod* function (Jenkinson et al., 2002; Tournier et al., 2004). Fiber tracking was performed for each subject using the *tckgen* function with the “iFOD2” option which performs improved second-order integration over fiber orientation distribution. This option enhances anatomical plausibility by facilitating more accurate fiber reconstruction in heavily curved regions (Tournier et al., 2004). During the tracking process, the probability of a particular direction is set to be proportional to the amplitude of the FOD along that direction. The following additional *tckgen* settings were used: max angle between successive steps = 22.5°, max length = 250 mm, min length = 10 mm, cut-off FA value = 0.4, and the maximum number of fibers = 1 million. The cut-off value for FA and max angle between successive steps are kept at a conservative level to ensure minimal false positives in the streamline estimation. The Region of Interests (ROIs) to extract the targeted interconnecting tracts were manually segmented by two independent observers and intersection of the two ROIs with overlap greater than 80% was considered as the final ROI. To extract the tract parameters, a fixel mask for the tracts was created using the *tck2fixel* function and applied over the whole brain FD (measure of density of intra-axonal space), FC (measure of cross-sectional size of the bundle in each voxel), FDC (the total capacity of the fiber bundle to carry information), and logFC fixel images of individual subjects after transforming them into a common template space.

### 2.5. Definition of regions of interest

ROIs were defined based on the automated anatomical labeling atlas 3 (AAL3; Rolls et al., 2020) and included the motor (region indexes: 1–2, 15–16, 61–62, 73–74) and prefrontal cortices (region indexes 19–20, 151–156). Masks of these regions were denormalized from MNI to individual native space using the inverse transform. For deep and small nuclei for which the inverse transform lacked spatial precision, we used a manual segmentation protocol. Two independent observers (V.R. and S.K.) draw the ROIs using FSLeyes (McCarthy, 2019), on the T1w MRI images (Thalamus and Putamen) and FOD images (STN and Dentate Nucleus). To control for inter-observer variability for each manual ROI, voxels that overlapped between the observers were considered for the analysis. We computed (i) the intersection volume of the ROIs by multiplying the binary mask of the ROI from two observers using *fslmaths and fslstats;* (ii) the total volume of their combination was calculated by adding them using *fslmaths and fslstats*. The ratio between the volume of intersection and combination of ROIs was computed. The overlap between each given ROI drawn by the two observers had to be greater than at least 80% to consider the ROI for the analysis.

#### 2.5.1. Thalamus

**Figure 2:**
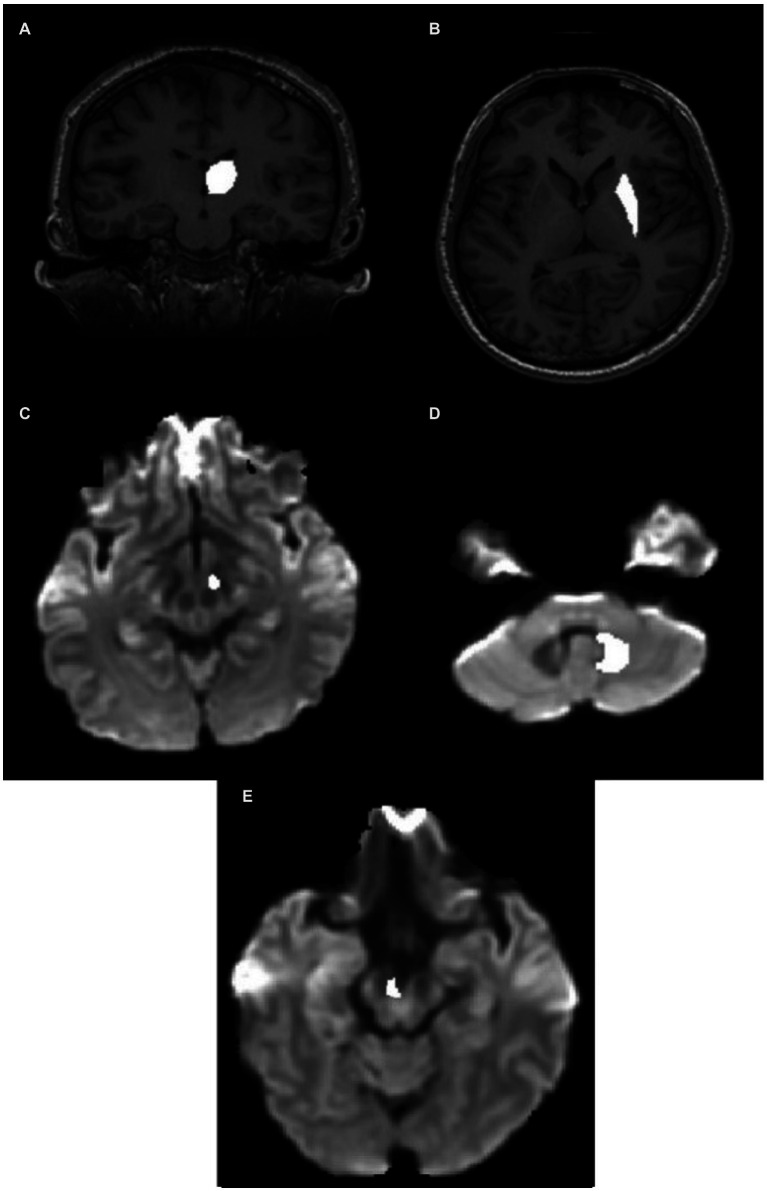
ROI definition in the individual native space. **(A)** Thalamic ROI mask superimposed on the T1 image. **(B)** Putamen ROI mask superimposed on the T1 image. **(C)** STN ROI superimposed on the Fiber Orientation Distribution (FOD) image. **(D)** Hypointense regions in the FOD image around the DN in the CB. **(E)** VTA ROI superimposed on the FOD image. CB, cerebellum; DN, dentate nucleus; STN, subthalamic nucleus; VTA, ventral tegmental area.

The thalamus was segmented on the T1-weighted image of each subject in the coronal plane (Figure 2A). The anterior boundary was the stria terminalis; the lateral and ventromedial boundaries were formed by the internal capsule; the genu and the caudate nucleus formed the dorsolateral boundary. Corrections were made in the sagittal and axial planes.

#### 2.5.2. Putamen

The putamen was segmented on the T1-weighted image of each subject in the axial plane (Figure 2B). The lateral border was the external capsule. The ventromedial boundary was the anterior limb of the internal capsule, whereas the posterior limb formed the posteromedial boundary. The mask was corrected for in the sagittal and coronal planes.

#### 2.5.3. Subthalamic nucleus

The STN (subthalamic nucleus) was segmented on the FOD image obtained from the diffusion-weighted image. It was located relative to the red nucleus, observable as the hypointense region at the brain stem as seen in the axial slice (Figure 2C). The STN was drawn 3 mm from the red nucleus in the anterolateral direction below the thalamus. The overlap between the manually drawn STN and standard atlas STN ROI denormalized to subject space is shown in Appendix Figure A1(B).

#### 2.5.4. Dentate

The DN (dentate nucleus) was drawn on the fiber orientation density image obtained from the diffusion-weighted image of individual subjects (Figure 2D) and defined by the hypointense semi-circular region in the CB with the opening facing the midline.

#### 2.5.5. Ventral tegmental area

VTA was segmented on the axial section of the FOD image obtained from the diffusion image as previously outlined by Ballard et al. (2011) as shown in Figure 2E. The overlap between the manually drawn VTA and standard atlas VTA ROI denormalized to subject space is shown in Appendix Figure A1(A).

### 2.6. Total intracranial volume

The total intracranial volume (TIV) was computed from the acquired T1-weighted images using Computational Anatomy Toolbox (CAT12) in the Statistical Parametric Mapping (SPM12) software running in MATLAB (R2019a: The MathWorks, Inc., Natick, Massachusetts, United States). The TIV value was calculated as the sum of volumes for White Matter (WM), Gray Matter (GM), and Cerebrospinal Fluid (CSF) obtained by segmenting the T1-weighted images into respective components.

### 2.7. Tract reconstruction

For all the ascending tracts described below, we considered the thalamus as an inclusion mask to build a reliable trajectory based on the anatomy of human and non-human primates (Hoshi et al., 2005; Bostan et al., 2010; Bostan and Strick, 2018). To identify the contralateral tracts, exclusion masks of the opposite cerebellar hemisphere and an interhemispheric mask covering the corpus callosum were considered.

#### 2.7.1. Cerebello-thalamocortical ascending tracts

Cerebello-thalamocortical (CTC) tracts projecting to cortical motor areas with known trajectories (Yamada et al., 2010) were studied as controls. We defined the cerebellar dentate nucleus as the seed region and the contralateral cortical motor areas as target regions (inclusion mask = contralateral thalamus).

#### 2.7.2. Cerebello-thalamo-striatal (CB-BG) ascending tract

We defined the dentate nucleus as the seed and the contralateral putamen as a target region (inclusion mask = contralateral thalamus).

#### 2.7.3. Subthalamo-cerebellar (a) crus II (b) VIIb descending tract

We defined the STN as the seed and the contralateral CrusII and VIIb as the target.

#### 2.7.4. Cerebello-prefrontal tracts *via* (a) VTA and (b) thalamus

Tractography was performed on tracts from the dentate nucleus to the contralateral prefrontal cortex (ACC/mPFC) with VTA (Carta et al., 2019) or thalamus as inclusion regions.

### 2.8. Statistical analysis

After verification of the normal distribution of data, to study the effect of age on morphological features of cerebellar tracts, a linear regression model was used to identify the association between individual mean parameters of FD, FC, and FDC across fixels of each tract (cerebello-thalamo-striatal and STN-Crus7b;) considering age as a covariable of interest, using the sex, education, and total intracranial volume (TIV) of the subjects as regressors of nuisance. The statistics and machine learning toolbox in MATLAB (R2019a: The MathWorks, Inc., Natick, Massachusetts, United States) was used for the analysis. Statistical significance was corrected for multiple comparisons using Bonferroni correction at *p* < 0.05. To study the relationship between the neuropsychological scores determined from ACE-M/RAVLT and FBA tract parameters, partial Pearson’s correlation analysis was performed after controlling for variables age, sex and education. Statistical significance was corrected for multiple comparisons using Bonferroni correction at *p* < 0.05.

### 2.9. Data availability statement

All the subject MRI data were collected at the Imaging Sciences and Interventional Radiology (IS/IR) Department and is the property of the Neurology Department at SCTIMST. These data will be made available to the academic researchers upon reasonable request to the corresponding author and the approval of the Institute Ethics Committee (IEC) at SCTIMST.

### 2.10. Declaration of competing interest

The authors declare that they have no known competing financial interests or personal ties that could have influenced the research presented in this study.

## 3. Results

Table 1 shows the demographics and the neuropsychological measures for the cognitive subject group.

**Table 1:** Table showing the demographics and the neuropsychological measures for the cognitive subject group.

Characteristic	Mean (*n = 20*)	Std. deviation
Age (years)	56.8	8.7
Sex (M/F)	11\09	
Education (years)	12.58	2.53
MMSE	29.1	0.95
ACE-M (Reg-24)^*^	21.86	1.58
ACE-M (Recall-10)^*^	7.52	1.53
ACE-M (total)	92.9	3
ACE-orientation	9.95	0.22
ACE-attention	7.95	0.22
ACE-memory	28.22	2.53
ACE-verbal fluency	13.6	0.66
ACE-visuospatial	4.6	0.74
ACE-language	27.85	0.65
RAVLT (total)*	47.24	8.08
RAVLT delayed recall	9.62	2.85

^*^ACE-M (Reg-24): Subdomain of ACE-Malayalam, registration/learning with 24-point scale (3 points for registration of 3 words and 21 points for learning of 3-trial learning of an address); ACE-M (Recall-10): subdomain of ACE-Malayalam with 10-point scale (3 points for recall of 3 words and 7 points for recall of address, each after a delay of 5 min); RAVLT, Rey Auditory Verbal Learning Test.

### 3.1. Tractography of CB-BG interconnecting networks

**Figure 3:**
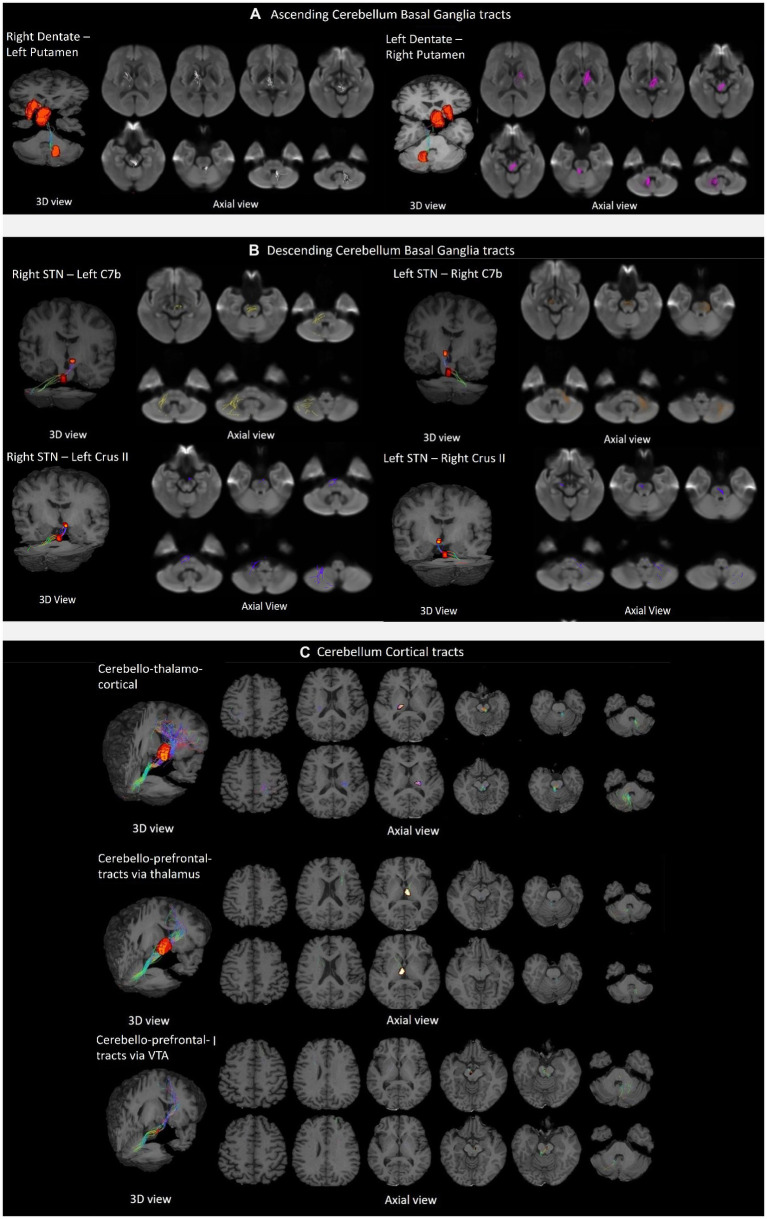
Tractography of crossed reciprocal tracts between the CB-BG and cortical targets in a representative subject. Tracts are superimposed on the study template (3D view, and axial views for a display of the tract trajectory). **(A)** Ascending cerebellum basal ganglia tracts; From left to right, Tracts between Right DN and Left Putamen, traversing Left Thalamus; Tracts between Left DN and Right Putamen, traversing Right Thalamus. **(B)** Descending cerebellum basal ganglia tracts; In the clockwise direction, Tracts between Right STN and contralateral Cerebellar cortex, C7b Left; Tracts between Left STN and contralateral Cerebellar cortex, C7b Right; Tracts between Right STN and contralateral Cerebellar cortex, C7b Left; Tracts between Left STN and contralateral CB cortex, Crus II Right; Tracts between Right STN and contralateral Cerebellar cortex, Crus II Left; **(C)** Cerebellar cortical tracts; From top to bottom, the first row shows the tracts between the DN and the sensorimotor cortex traversing the ventral intermediary nucleus of the thalamus. The top and bottom rows display axial views of the right CB-left thalamus/Cortex and left CB-right Thalamus/Cortex respectively; The second row shows the tracts between the DN to the contralateral mPFC/ACC *via* the medial dorsal nuclei of the thalamus. The top and bottom rows display axial views of the left CB-right Thalamus/Cortex and right CB-left thalamus/Cortex, respectively. The third row shows the tracts from the dentate to the contralateral mPFC/ACC *via* the ventral tegmental area.

The tractography analysis revealed the bilateral and reciprocal tracts between the CB and BG. The ascending tract between the dentate nucleus to the contralateral thalamus traversed the superior cerebellar peduncles and decussated at the level of the midbrain to the contralateral side, terminating in the Putamen (Figure 3A). The descending tract between STN to the contralateral cerebellar cortex, C7b and Crus II, traversed the basilar part of the pons, decussated along regions associated with the pontine nuclei, and passed through the middle cerebellar peduncle (Figure 3B).

We generated a specific fixel mask for each of the ascending and descending tracts (in the study template space) using the individual white matter FOD image that was spatially normalized in the study template space. The fixel mask generated from the tracts was intersected with the whole brain white matter FD, FC, and FDC fixel data image to obtain the tract-specific metric for individual subjects.

### 3.2. Tractography on the control tracts

**Figure 4:**
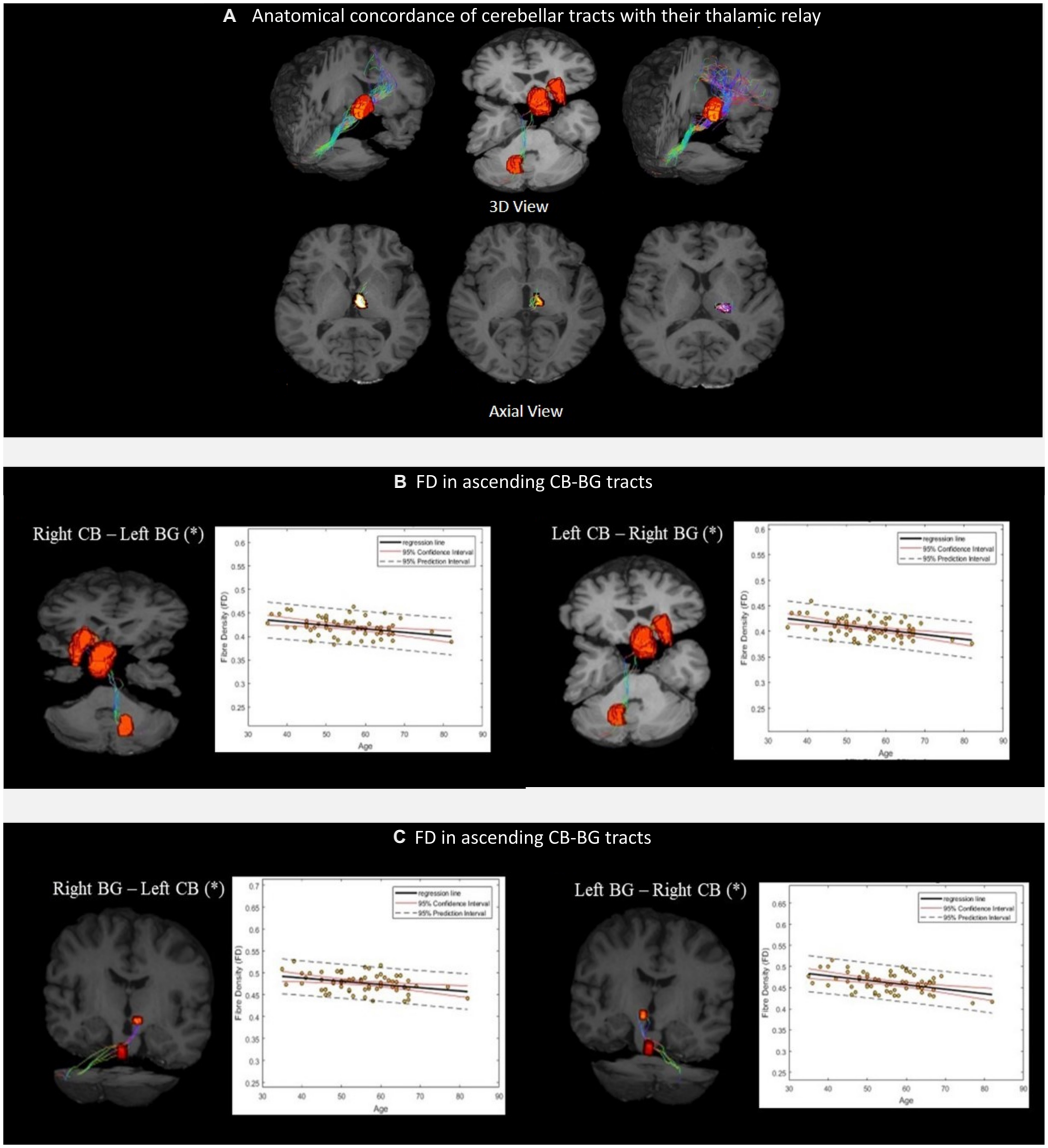
**(A)** Thalamic nuclei as specific relays of the cerebellar tracts. From left to right, Medial Dorsal (MD) nuclei with cerebello-frontal tracts; Central median (CM) nuclei with cerebello-thalamo-striatal (CTS) tracts; Ventral Intermediary (VIM) nuclei with cerebello-thalamocortical (CTC) tracts. Scatterplots **(B,C)** show the mean FD values of the corresponding CB-BG tracts included in the analysis. The black solid lines represent the regression line, whereas the dashed red lines represent the 95% confidence interval and the black dashed lines represent the 95% prediction interval. (*) indicates the significant linear relationship with age.

We successfully reconstructed the CTC tracts (Figure 3C). For the sensorimotor tracts, the DN exited the CB *via* the superior cerebellar peduncle, decussated at the level of the midbrain, traversed the contralateral thalamus *via* the ventral intermediary nucleus (VIM), and reached the contralateral primary motor and sensory cortices (Figure 3C). For the associative tracts, the dentate exited the CB *via* the superior cerebellar peduncle, traversed the contralateral thalamus *via* the mediodorsal nuclei, and reached the contralateral frontal cortex (ACC/mPFC; Figure 3C). In addition, we reconstructed the tracts between the dentate nucleus to the contralateral frontal cortex (ACC/mPFC) passing through the VTA (Figure 3C). The reconstruction of the cerebellar tract was performed using an inclusion mask comprising the whole thalamus. However, looking at the tract trajectories through their thalamic relays, (i) the CB-BG tract passed through the central-medial nucleus; (ii) the CTC connecting the sensorimotor areas passed through the ventral intermediary nucleus; (iii) the CTC connecting the prefrontal areas passed through the medial-dorsal nucleus. These thalamic relays (Figure 4A) are in correspondence with what is known of the neuroanatomy of these pathways.

The linear regression analysis showed a significant negative linear relationship between FD and age for the ascending and descending CB-BG tracts (Figures 4B, C; Table 2). In all the tracts, the mean FC and logFC values were unaffected by age. FDC values were found to be negatively correlated with age in the ascending tract between the right Dentate to left Putamen.

**Table 2:** Table showing the estimated value of the standardized regression coefficients for the tract-level analysis metrics along with their *p*-values.

FBA metric	Tracts	Age		Sex		TIV		Education	
		β	p	β	p	β	p	β	p
FD	Dn_L → Put_R	**−0.40994**	**0.00087457**	−0.03281	0.82751	−0.18287	0.2317	0.190501	0.10523
	Dn_R → Put_L	**−0.49802**	**5.26E-05**	0.01276	0.93086	−0.1371	0.35745	0.097072	0.39419
	STN_R → C7b_L	**−0.35857**	**0.00024516**	0.324362	0.59763	−0.23155	0.83761	0.184973	0.5422
	STN_L → C7b_R	**−0.4552**	**0.0031548**	0.079334	0.053923	−0.03106	0.12995	0.070781	0.11418
FDC	Dn_L → Put_R	−0.25413	0.0624673	−0.11796	0.40737	**0.502846**	**0.00083**	0.075748	0.4905
	Dn_R → Put_L	**−0.29739**	**0.0054045**	−0.09823	0.45983	**0.563221**	**8.36E-05**	−0.01399	0.89133
	STN_R → C7b_L	−0.10082	0.078241	0.124569	0.65756	**0.394158**	**0.00022**	0.114996	0.69805
	STN_L → C7b_R	−0.22577	0.37206	−0.06085	0.38969	**0.542427**	**0.00865**	0.041124	0.30452
log_FC	Dn_L → Put_R	−0.20606	0.1115	−0.11263	0.49373	−0.09478	0.56809	0.060059	0.6362
	Dn_R → Put_L	−0.13495	0.29755	−0.1608	0.33275	−0.06666	0.68981	0.050139	0.69481
	STN_R → C7b_L	0.128051	0.58676	−0.12466	0.26621	**0.507762**	**0.00575**	0.019757	0.74253
	STN_L → C7b_R	0.067096	0.28536	−0.17672	0.4164	**0.455513**	**0.00163**	0.040131	0.86719

The values of regression coefficients in bold are significant at Bonferroni corrected *p*-value of *p* < 0.05.

Mean values of FDC across fixels within all the tracts showed a significant positive linear relationship with TIV (Table 2). TIV was also found to have a significant positive linear relationship with the mean parameter, log_FC of the bilateral tract from STN to the Cerebellar cortex. TIV was significantly higher in males compared to females, most likely reflecting a gender effect. Multiple regression analysis of gray matter volume with age considering education, TIV, and sex as covariates yielded no significant relation at FWE corrected *p*-value of 0.05.

### 3.3. Neuropsychological analysis

#### 3.3.1. Ascending tracts

**Figure 5:**
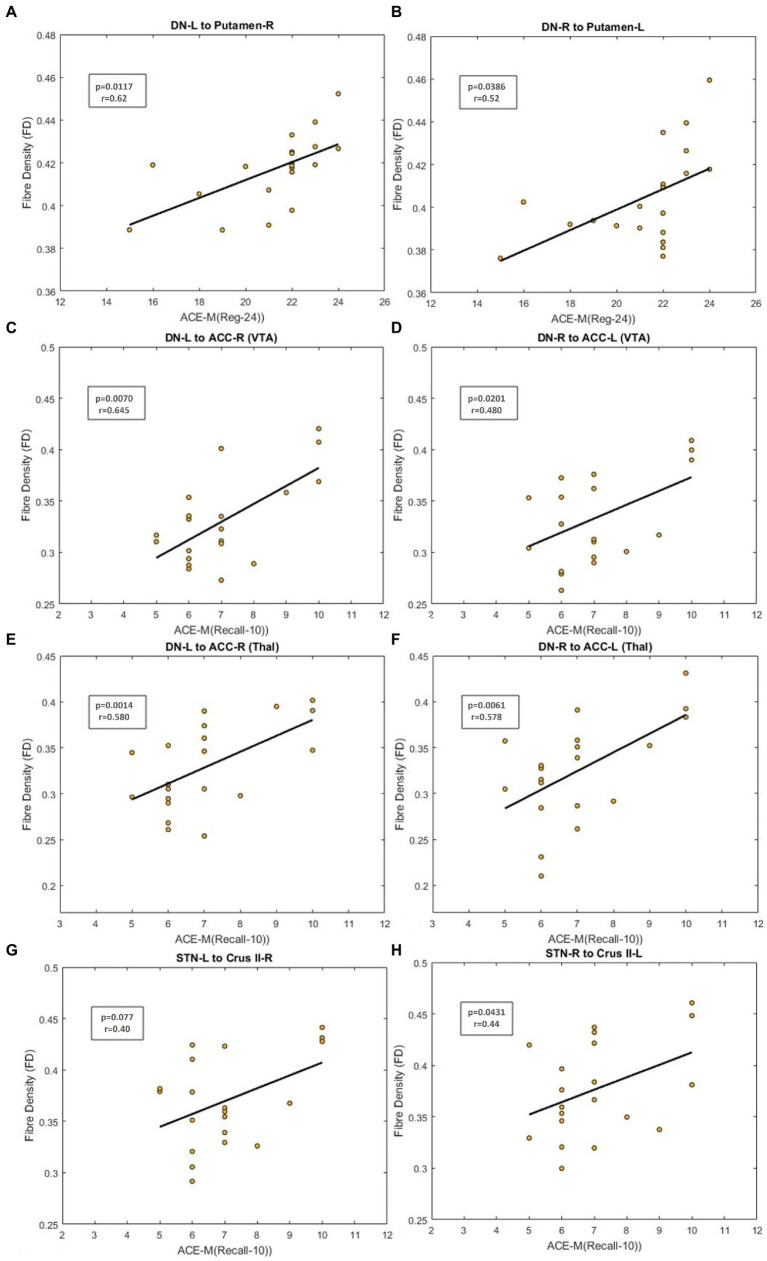
Figure showing the scatterplot between Fiber Density (FD) and neuropsychological scores. **(A,B)** Scatterplot for ACE-M (Reg-24) against FD for the dentate nucleus to contralateral Putamen tract. **(C,D)** Scatter plot for ACE-M (Recall-10) against FD for the dentate nucleus to prefrontal cortex tract *via* VTA **(E,F)** Scatter plot for ACE-M (Recall-10) against FD for the dentate nucleus to prefrontal cortex tract *via* thalamus **(G,H)** Scatter plot for ACE-M (Recall-10) against FD for tracts from STN to contralateral Crus II tract.

FD in the cerebello-thalamo-striatal tracts was positively correlated with the ACE-M (Reg-24) score (DN_L to Putamen_R, p_corrected_ = 0.0117; DN_R to Putamen_R, p_corrected_ = 0.0386 as in Figures 5A, B). For the DN-mPFC tracts passing through thalamic or VTA relays, FD was positively correlated with the ACE-M (Recall-10) score (DN_L-VTA-ACC_R, p_corrected_ = 0.0070; DN_R-VTA-ACC_L, p_corrected_ = 0.0201 as in Figures 5C, D; DN L-Thal-ACC_R, p_corrected_ = 0.0014; DN_R-Thal-ACC_L p_corrected_ = 0.0061 as in Figures 5E, F). The RAVLT, ACE-M (total), and ACE-M (Reg-24) scores did not correlate with FD in any of these tracts. In the case of CTC tracts, none of the tract parameters correlated with the neuropsychological parameters. Other subdomains of ACE-M were not found to be significant with any of the FBA metric of the ascending tracts.

#### 3.3.2. Descending tracts

The FBA metrics for the tracts from STN to the contralateral cerebellar cortex were extracted for STN-C7b tracts as well as STN-CrusII tracts. Pearson’s correlation between the FBA metrics of the C7b tract and neuropsychological scores of ACE-M and RAVLT did not yield any significant relationship. FD in the STN-R_CrusII_L was positively correlated with ACE-M(Recall-10) score (p_corrected_ = 0.0431) as in Figures 5G, H. The ACE-M, ACE-M (Reg-24), ACE-M (total), RAVLT, and RAVLT Delayed Recall scores did not correlate with FD in this tract. Other subdomains of ACE-M were not found to be significant with any of the FBA metric of the descending tracts.

## 4. Discussion

We validated the subcortical CB-BG connection in a large database of 64 healthy volunteers and expanded the findings reported earlier in primates and small samples of healthy subjects. In addition, we showed for the first time, that the microstructure of subcortical reciprocal connections between the CB and BG in human subjects was affected by aging. We further explored the cognitive functional roles of these tracts and found that inter-individual variability of descending and ascending CB-BG reciprocal tracts was associated with cognitive scores. These findings bring important consideration for the understanding of neurodevelopmental and neurodegenerative diseases.

### 4.1. White matter tracts constituting the CB-BG direct subcortical network

The tractography using constrained spherical deconvolution that addresses the issue of crossing fibers present in the majority of the white matter voxels (Raffelt et al., 2015), validated the presence of ascending tracts from the output nuclei of the CB, the dentate nucleus, to the contralateral putamen, *via* the thalamus. The tracts traversed primarily through the centromedian nucleus of the thalamus before reaching the putamen (Figure 4A). This is an important validation since the connections from the dentate to the striatum in mice pass through the anatomical equivalent thalamic nucleus (Coutant et al., 2022). A similar trajectory of the dentato-thalamo-striatal pathway through the thalamus was reported in the transneuronal viral transport study in macaques (Hoshi et al., 2005).

The descending tracts from the STN were found to innervate the contralateral cerebellar cortex *via* the middle cerebellar peduncles with decussations along the regions that include the pontine nuclei (Figure 3B). We showed that the CB-BG subcortical tracts in healthy human subjects have similar trajectories as reported in non-human primate studies and is congruent with the findings of Milardi et al. which established the dentato-thalamic pathways (Milardi et al., 2016).

We performed tractography on the cerebello-thalamo-cortical tract as a control tract with a known trajectory (Yamada et al., 2010) from the dentate nucleus to the primary motor and sensory cortical areas. We confirmed that this tract passes *via* the VIM nucleus of the thalamus, thus establishing that the tractography algorithms used in the study have anatomical validity. In addition, we showed that CB tracts also reach ACC, and mPFC, passing through the VTA and medial-dorsal nucleus of the thalamus.

The projections from VTA to the prefrontal cortex (Björklund and Dunnett, 2007) mediate higher-order cognitive functions, and the recent work of Carta et al. (2019) proves the role of cerebellum-VTA connection in social behavior and reward circuitry. We, therefore, tested for a direct connection from cerebellar output nuclei to the prefrontal cortex *via* VTA. The tractography results in Figure 3C demonstrate the anatomical connections from the dentate nucleus to the prefrontal cortex *via* VTA.

### 4.2. Age-related changes in the morphometry of the CB-BG connections

Going further than the anatomical description of the tracts, we demonstrated for the first time, a negative linear relationship between age and FD in the ascending and descending tracts between CB-BG. Fiber morphometric measures are an indicator of the ability of the axonal bundle to relay information, depending on the number of axons and the volume of the axonal cross-section. In the FBA analysis technique used in this study, the FD metric, calculated as an integral of the fiber orientation distribution in a fixel, is proportional to the intra-axonal volume of the fiber bundles in that fixel and is a measure of the number of axons in a fiber bundle (Raffelt et al., 2012). The relationship between FD and age in CB-BG reciprocal tracts could arise from reduced free water volume in the fixel associated with aging and possibly axonal loss.

Age-related changes to whole-brain white matter morphology have been widely reported both in human and animal studies using the diffusion scalar metrics such as Fractional Anisotropy (FA), Radial Diffusivity (RD), and Axial Diffusivity (AD) as well as postmortem studies (Tang et al., 1997; Salat et al., 2005; Bowley et al., 2010; Sala et al., 2012; Bennett and Madden, 2014; Cox et al., 2016). However, very few studies have investigated precise cerebellar tracts. The age-related decline in FD indicates a reduction in the number of fibers in the white matter bundle connecting the CB-BG with advancing age. Postmortem studies on age-related WM atrophy in human subjects at the corpus callosum have identified a reduction in the number of fibers and density as the primary factor contributing to WM atrophy (Hou and Pakkenberg, 2012). FD and FC provide a macrostructural and microstructural measure of change in the fiber tracts, respectively, and their combined measure of FDC obtained as a product of FD and FC provides an overall measure of the ability of the fiber bundle to relay information (Raffelt et al., 2017). One of the arguments proposed to explain the relative preservation of FC with age is the presence of different caliber axons in the bundle. The larger axons are better preserved during aging, thus maintaining FC (Choy et al., 2020). and this could explain preserved FC in the CB-BG connections in this study. Greater axonal caliber is associated with a higher information rate (Perge et al., 2012). The preservation of FC in the CB-BG with aging could indicate these tracts may be involved in the rapid information exchanges that are necessary to efficiently update the comparison between expectations with the current state of the cognitive and motor system, a function that is dear to the cerebellum. Future studies including more of older subjects are required to quantify the relation between age-related deterioration in motor function and FC of the CB-BG network.

To fully comprehend the functional changes associated with the age-related degeneration of the CB-BG tracts, it is imperative to understand the changes in BG as well as thalamic regions. The striatal regions of the putamen and caudate undergo bilateral shrinkage with age and their volumes display a negative trend with age (Gunning-Dixon et al., 1998; Raz et al., 2003; Koikkalainen et al., 2007). Similarly, STN volumes and cell count also decrease in an age-dependent fashion (Zwirner et al., 2016). In the case of the thalamus which forms the relay between the CB and BG regions, the volume also has a downward trend with aging (Pfefferbaum et al., 2013). The regions of the brain that age first and result in changes in the FD of CB-BG tracts are yet to be identified.

### 4.3. Psychometric and behavioral correlations with CB-BG tract microstructure

We found a positive association between FD of subthalamo-cerebellar and cerebello-frontal tracts and the learning/retention domain of ACE-M scoring. Descending tracts between STN to CrusII have been primarily associated with non-motor processes (Guell and Schmahmann, 2020). Viral tracing studies on non-human primates demonstrated the presence of second-order neurons projecting from CrusIIp to STN (Bostan et al., 2010). In addition, the basic framework for a resting-state executive control network consists of regions from BG such as the associative territory of the striatum, the caudate, and regions of CB such as Crus I and II along with the prefrontal cortical region and is associated with non-motor functions such as verbal fluency and working as well as episodic memory (Habas et al., 2009). The relationship we show between the tract FBA metrics and cognitive scores involves the cerebello-frontal network and reciprocal CB-BG network. Subthalamo-cerebellar tract from STN to pontine nuclei to Crus II showed a positive linear relationship with the FD metric while the FBA metrics of the subthalamo-cerebellar tract to C7b showed no significant linear relationship with any of the neuropsychological scores. This is congruent with the fact that the CB C7b region is primarily associated with motor tasks and CrusII is primarily involved in non-motor functions (Van Overwalle et al., 2020a,b).

We found that individual neuropsychological scores were correlated with individual FD values in tracts from the CB to ACC/mPFC region traversing (a) VTA and (b) mediodorsal nuclei of the thalamus. FD for these tracts had a positive linear relationship with the 10-point score of 10 min recall of 3 words and address, ACE-M (Recall-10). Lower FD values could be associated with a reduction in the information-carrying capability of the white matter bundle leading to a reduction in the ability to recall the word list and address from memory and *vice-versa*. In the case of the cerebello-thalamo-striatal tract (putamen), FD had a positive linear relationship with the ACE-M (Reg-24) scores, implying that this tract could be primarily associated with the initial learning capability of the words/address. Neuroimaging studies on instrumental learning have demonstrated the role of putamen in the acquisition of cue-response association during the exploration phase (Brovelli et al., 2011). This could imply the cerebello-thalamo-putamen tract properties can affect learning and memory registration.

### 4.4. Implication for movement disorders where BG and CB are affected

Connections between CB and BG are particularly relevant to BG disorders such as PD and dystonia (Wu and Hallett, 2013; Bologna and Berardelli, 2017). Supporting findings for the presence of the dentato-thalamo-striatal pathway in human subjects come from deep brain stimulation (DBS) studies on dystonia and tremor patients (Paraguay et al., 2021). Despite being conventionally classified as a BG disorder, in PD patients with tremor symptoms, DBS of the thalamic nuclei, ventral intermediary nuclei (VIM), which receive input from the CB, provides greater beneficial effects rather than the nuclei receiving inputs from BG (Narabayashi et al., 1987). Preliminary finding on DBS of the dentate nucleus has shown positive outcomes for dystonia and tremor patients (Diniz et al., 2021). The STN is an excitatory nucleus that drives the output of the BG. Thus, age-dependent structural changes of CB-BG tracts might influence the efficacy of STN-DBS.

In the reciprocal connections of the CB-BG tract, little is known about the functional involvement of feedback loops connecting the cerebrum to the cerebellum through the STN.

Basal ganglia hyperactivity in PD patients was supposedly transferred to the cerebellum *via* subthalamo-cerebellar connections (Asanuma et al., 2005) and STN-DBS ameliorates the hyperactivity in the CB thereby improving motor function in PD patients (Payoux et al., 2004; Asanuma et al., 2006). Age-related progressive loss in nigrostriatal dopamine neurons has been recorded to result in age-related functional deficits (Gibb and Lees, 1991). A multi-fold acceleration of this phenomenon leads to PD. Similarly, it can be hypothesized that over time, an accelerated loss of CB-BG subcortical tracts as demonstrated in aging could substantially weaken the cerebellar control over BG and contribute to the pathophysiology of PD. In the case of spinocerebellar ataxias (SCA), which are clinically and genetically heterogeneous, the mean age at onset of symptoms differs for SCA1, SCA2, SCA3, SCA6, SCA7, and could vary from third to fourth and even sixth decade of life (Warrenburg et al., 2005). The knowledge of the age effects of CB-BG circuits could help better understand the clinically diverse manifestation of the disease including the occurrence of parkinsonism and dystonia in some of the SCA (Meira et al., 2019).

### 4.5. Limitations

One of the limitations of this study is the use of a single shell and relatively low b-value (b = 1000S/mm^2^) for the acquisition of diffusion data. Estimation of FOD and apparent FD (AFD) could be made more robust with higher and multiple b-value acquisitions. Another limitation of the study is the medium range of the effect size (Cohen’s f^2^: 0.15–0.35) for the multiple linear regression analysis for age vs. FD. Even though the negative linear relation is statistically significant, the change in FD with unit change in age is smaller. Another limitation is the small sample size of cognitive data. Although the results provide a new perspective on the functional role of these tracts in memory registration and recall, a longitudinal study with a larger sample size is required to further validate the results of the association between CB-BG tract parameters and cognitive scores. Even though the correlation values between the neuropsychological scores and FD values provide information on the involvement of these tracts on the task, the exact role played by these tracts in the memory and learning network needs to be further explored.

## 5. Conclusion

In this study, we confirmed the existence and trajectories of subcortical connections between the CB and BG in a large group of human subjects, as reported in non-human primates. We observed that FD in reciprocal CB-BG tracts was negatively correlated with age and positively correlated with specific cognitive psychometric scores. The recall memory assessment domain of ACE-M was associated with FD for both the subthalamic-cerebello (Crus II) tract and the cerebello-frontal tracts to the prefrontal cortex. The FD metric in the cerebello-thalamo-putamen tracts was positively correlated with the learning/registration domain of the ACE-M score. Further investigation is required to study the functional roles of these tracts in movement disorders particularly PD and dystonia and its therapeutic implications. Deepening our knowledge of the functional neuroanatomy of the CB-BG connections in humans has much value in understanding their interactions in health and disease. This warrants more studies, including post-mortem studies, for confirmation of these interconnections.

## Data availability statement

The datasets presented in this article are not readily available because of ethical and privacy restrictions. Requests to access the datasets should be directed to asha.kishore@asterdmhealthcare.com.

## Ethics statement

The studies involving human participants were reviewed and approved by Institutional Ethics Committee, Sree Chitra Tirunal Institute for Medical Sciences and Technology, Kerala, India. The patients/participants provided their written informed consent to participate in this study.

## Author contributions

AK, SK, CG, and VR formulated the research goals. AK acquired funding and supervised the project. VR, PJ, and RM acquired the data. VR, CG, and RV analyzed the data. VR, AK, and CG wrote the manuscript. SK, CK, and BT read and revised the manuscript. All authors contributed to the article and approved the submitted version.

## Funding

This work received intramural funding (Project No: 5170) from the Sree Chitra Tirunal Institute for Medical Sciences and Technology, Kerala, India.

## Conflict of interest

The authors declare that the research was conducted in the absence of any commercial or financial relationships that could be construed as a potential conflict of interest.

## Publisher’s note

All claims expressed in this article are solely those of the authors and do not necessarily represent those of their affiliated organizations, or those of the publisher, the editors and the reviewers. Any product that may be evaluated in this article, or claim that may be made by its manufacturer, is not guaranteed or endorsed by the publisher.
