# Supplementary figures and images for: Cerebellar and basal ganglia structural connections in humans: Effect of aging and relation with memory and learning
Source: Front Aging Neurosci. 2023 Jan 26;15:1019239. doi: 10.3389/fnagi.2023.1019239 (PMC9908607; doi:10.3389/fnagi.2023.1019239)

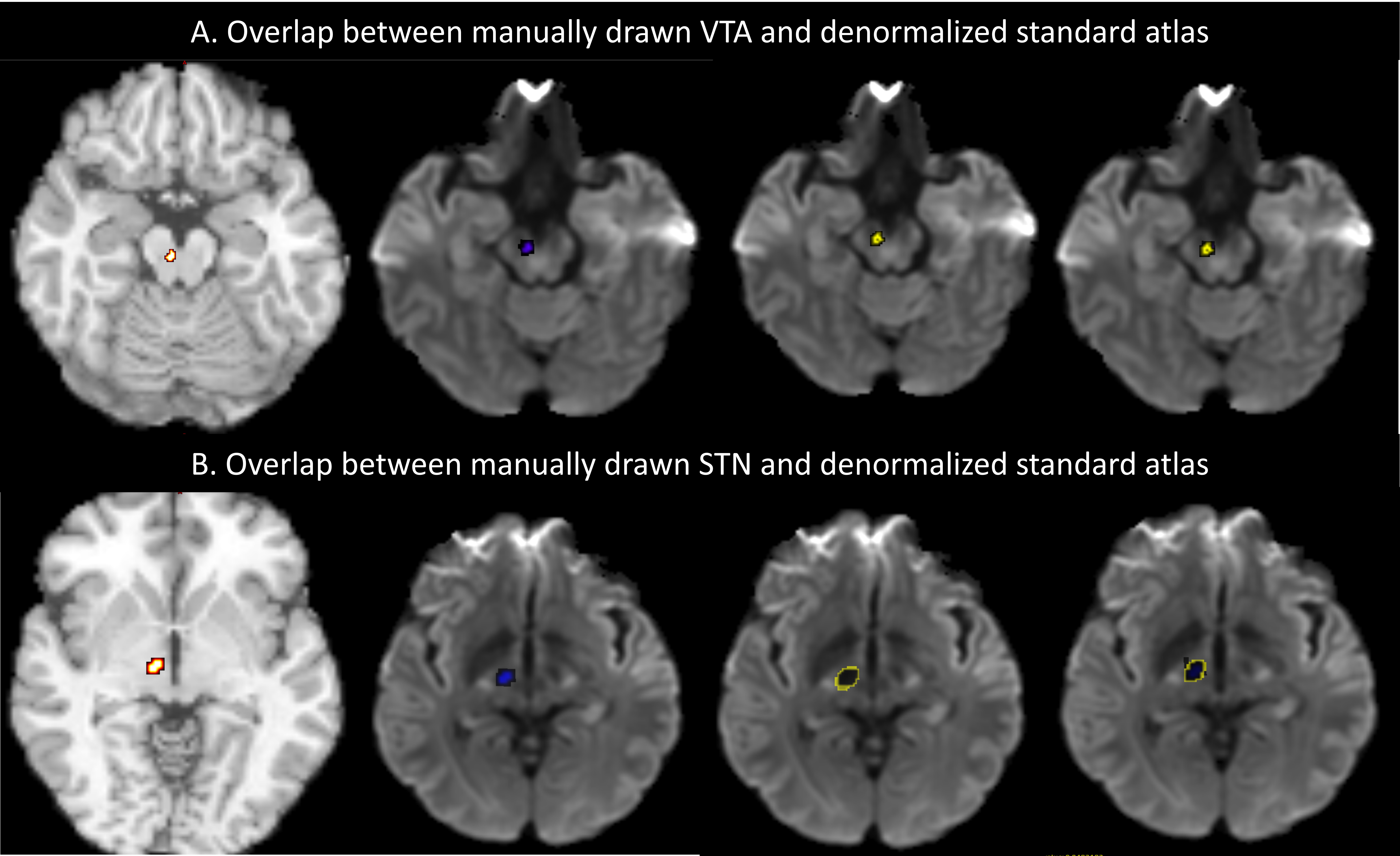

Supplement: Supplementary file 1 [file Image_1.JPEG]
