# Supplementary material for: Cerebellar and basal ganglia structural connections in humans: Effect of aging and relation with memory and learning
Source: Front Aging Neurosci. 2023 Jan 26;15:1019239. doi: 10.3389/fnagi.2023.1019239 (PMC9908607; doi:10.3389/fnagi.2023.1019239)
Supplement: Supplementary file 2 [file Image_2.pdf]

Figure A1. Figure showing the overlap between the manually drawn A) VTA ROI and B) STN ROI and standard atlas ROIs denormalized to the subject space. The figure shows, from left to right, the standard atlas ROI in MNI space, the atlas ROI normalized to subject space, the manually drawn ROI in subject space, the overlap of the denormalized standard atlas, and the manually drawn ROI in subject space.
